# Nitrofurantoin Combined With Amikacin: A Promising Alternative Strategy for Combating MDR Uropathogenic *Escherichia coli*

**DOI:** 10.3389/fcimb.2020.608547

**Published:** 2020-12-21

**Authors:** Zi-Xing Zhong, Ze-Hua Cui, Xiao-Jie Li, Tian Tang, Zi-Jian Zheng, Wei-Na Ni, Liang-Xing Fang, Yu-Feng Zhou, Yang Yu, Ya-Hong Liu, Xiao-Ping Liao, Jian Sun

**Affiliations:** ^1^National Risk Assessment Laboratory for Antimicrobial Resistance of Animal Original Bacteria, South China Agricultural University, Guangzhou, China; ^2^Guangdong Provincial Key Laboratory of Veterinary Pharmaceutics Development and Safety Evaluation, South China Agricultural University, Guangzhou, China; ^3^Department of Laboratory Medicine, The Third Affiliated Hospital, Sun Yat-sen University, Guangzhou, China; ^4^Guangdong Laboratory for Lingnan Modern Agriculture, Guangzhou, China

**Keywords:** MDR UPEC, nitrofurantoin, amikacin, antibiotic combination, *G. mellonella* model

## Abstract

Urinary tract infections (UTI) are common infections that can be mild to life threatening. However, increased bacterial resistance and poor patient compliance rates have limited the effectiveness of conventional antibiotic therapies. Here, we investigated the relationship between nitrofurantoin and amikacin against 12 clinical MDR uropathogenic *Escherichia coli* (UPEC) strains both *in vitro* and in an experimental *Galleria mellonella* model. *In vitro* synergistic effects were observed in all 12 test strains by standard checkerboard and time-kill assays. Importantly, amikacin or nitrofurantoin at half of the clinical doses were not effective in the treatment of UPEC infections in the *G. mellonella* model but the combination therapy significantly increased *G. mellonella* survival from infections caused by all 12 study UPEC strains. Taken together, these results demonstrated synergy effects between nitrofurantoin and amikacin against MDR UPEC.

## Introduction

Urinary tract infections (UTI) are defined microbiologically as the inflammatory response of the urothelial to microbial pathogens and are some of the most common bacterial infections affecting 150 million people each year worldwide (Klein and Hultgren, 2020). UTIs are most commonly associated with uropathogenic *Escherichia coli* (UPEC) and *E. coli* ST131 is the globally dominant multiple drug-resistant (MDR) UPEC clone that causes infections associated with limited treatment options (Daoud et al., 2015; Phan et al., 2020). These infections can also be highly recurrent and following antibiotic therapy, 20–30% of women with acute UTIs will have a recurrent episode within six months and half of these recurrences are caused by the same UPEC strain that caused the initial infection (Godaly et al., 2015). The increases in bacterial resistance as well as poor patient compliance rates have limited the effectiveness of conventional antibiotic therapies for UTIs especially in developing countries (Ayukekbong et al., 2017; Goodlet et al., 2018). Therefore, there is a great need for alternative strategies to treat these infections and one important approach is combination therapy using pre-existing antibiotics (Brochado et al., 2018).

Carbapenems have been recommended for treating UTIs caused by extended spectrum β-lactamase (ESBL) producing bacteria but currently are being restricted due to increased resistance (Han et al., 2015). However, non-carbapenem antibiotics are also important for UTI treatment. Trimethoprim-sulfamethoxazole (TMP-SMX) was the preferred antibiotic for UTI treatment for many years due to its efficacy and low cost. However, the development of TMP-SMX resistance among uropathogens has altered this strategy and fluoroquinolones are now preferred because they are highly concentrated in urine and have excellent activity against most uropathogens (Johnson and Stamm, 1987). The US Food and Drug Administration (FDA) approved nitrofurantoin in 1953 and was the standard UTI treatment until the late 1970s when other antibiotics became available (Muller et al., 2017). In 2011 nitrofurantoin was again recommended as first-line therapy for lower UTI due to increasing resistance to newer antibiotics such as the fluoroquinolones (Gupta et al., 2011). Antibiotic resistance is spreading rapidly in UPEC, which may be related to the genetic modulate, own pathogenicity and drug resistance of themself. The use of fluoroquinolones can induce the UPEC to partial or total loss of the pathogenicity islands and lead to cross-resistance of β-lactam drugs (Soto et al., 2006; Rohde et al., 2018; Adamus-Bialek et al., 2019; Tchesnokova et al., 2019). On the other hand, the high prevalence of integrons and plasmids also leads to high levels of antibiotic resistance and virulence genes in clinical urogenic bacteria, such as resistance of extended spectrum β-lactamase (ESBL) producing and to quinolones (Raeispour and Ranjbar, 2018; Abbasi and Ranjbar, 2018; Farajzadah Sheikh et al., 2019; Halaji et al., 2020a; Halaji et al., 2020b). However, the use of nitrofurantoin is contraindicated in patients with renal failure due to metabolites that may cause peripheral neuropathy (Spring et al., 2001). Similarly, nitrofurantoin is not recommended for the treatment of complicated UTIs because these infections often compromise the kidney and lead to renal dysfunction (Ingalsbe et al., 2015). In contrast, amikacin is a first-line drug used for Gram-negative infections other than UTIs that is economical and convenient to administer. Its potential ototoxicity and nephrotoxicity is dose related so that it could be efficacious and safe for the treatment of pyelonephritis and sepsis if managed properly (Leibovici et al., 2009).

Interestingly, amikacin and nitrofurantoin can synergize against *E. coli in vitro* (Yeh et al., 2006), and these two antibiotics are used separately to treat or prevent UTIs caused by MDR *E. coli*. In this study, we examined whether the co-administration of amikacin and nitrofurantoin could provide a new strategy for UTI treatment and evaluated the synergistic effects of these two drugs *in vitro* and *in vivo*.

## Materials and Methods

### Bacterial Strains and Culture Conditions

Twelve clinical isolates were obtained from the urine of hospitalized UTI patients at the Third Affiliated Hospital of Sun Yat-Sen University (Guangzhou, China). All tested strains were identified to the species level by MALDI-TOF MS (Axima-Assurance-Shimadzu) and *E. coli* ATCC 25922 was used as a quality control strain (**Table 1**).

**Table 1:** *In vitro* antimicrobial susceptibility profiles for clinical strains.

E. coli strain	Relevant genotype	MIC (mg/L)										
		NIF	AMK	MEM	AMP	CTX	FOS	CST	TIG	TET	CIP	SXT
ATCC 25922	ST73	16	4	0.03	4	0.125	2	2	0.06	1	0.015	1/19
E7102	ST131	16	2	0.015	>256	256	0.25	2	0.06	64	128	>16/304
E6929	ST131	16	4	0.25	>256	>256	0.5	1	2	1	128	>16/304
E4396	ST1193	16	2	0.015	>256	>256	2	4	0.06	64	32	>16/304
E3759	ST101	16	4	0.03	>256	>256	64	0.5	0.25	128	16	>16/304
E7088	ST1426	16	2	0.015	>256	>256	4	2	0.125	256	1	>16/304
E4181	ST53	16	4	0.015	>256	256	8	2	0.06	2	32	>16/304
E68071	ST3177	8	2	0.015	>256	256	16	2	0.03	64	128	>16/304
E67991	ST3177	16	2	0.25	>256	>256	0.25	2	2	1	64	>16/304
E3966	ST354	8	4	0.015	>256	>256	>256	0.5	0.03	128	>256	>16/304
E4740	ST53	16	4	0.015	>256	>256	8	1	0.06	64	32	>16/304
E68317	ST1249	16	16	0.015	>256	>256	256	2	0.125	64	2	>16/304
E62603	ST1196	32	4	0.125	>256	>256	8	0.5	4	128	64	>16/304

NIF, nitrofurantoin; AMK, amikacin; MEM, meropenem; AMP, ampicillin; CTX, cefotaxime; FOS, fosfomycin; CST, colistin; TIG, tigecycline; TET, tetracycline; CIP, ciprofloxacin; SXT, sulfamethoxazole/trimethoprim.

Genomic DNA from the 12 clinical isolates was subjected to 250 bp paired-end whole genome sequencing (WGS) using the Illumina MiSeq system (Illumina, San Diego, CA, USA) and the reads were assembled using SPAdes v3.6.2. (Bankevich et al., 2012) MLST and antibiotic resistance genes (ARG) were analyzed using the CGE server (https://cge.cbs.dtu.dk/services/) and ABRicate (https://github.com/tseemann/abricate).

### Antimicrobial Agents

Amikacin (AMK), nitrofurantoin (NIF), meropenem (MEM), cefotaxime (CTX), tigecycline (TIG) and tetracycline (TET) were purchased from Yuan Ye Biological Technology (Shanghai, China). Ampicillin (AMP), fosfomycin (FOS), colistin (CST), ciprofloxacin (CIP) and sulfamethoxazole/trimethoprim (SXT) were purchased from Xiang Bo Biotechnology (Guangzhou, China). Antibiotic stocks solutions were prepared according to the manufacturer’s recommendations.

### MIC Determinations

Antimicrobial susceptibility assays were performed and interpreted according to CLSI guidelines (CLSI, 2018) using the microdilution broth method except for fosfomycin. The MIC of Fosfomycin was tested using the agar dilution method in agar media supplemented with 25 mg/L glucose-6-phosphate. *E. coli* strain ATCC 25922 was used for quality control.

### *In Vitro* Fractional Inhibitory Concentration Index (FICI) Assay

The checkerboard technique was employed to determine the Fractional Inhibitory Concentration Index (FICI) of nitrofurantoin/amikacin combinations as previously described (White et al., 1996). Briefly, 96 well plates containing serial dilutions of nitrofurantoin and amikacin (range 0.125 to 32 mg/L) were inoculated with 5 × 10 (Goodlet et al., 2018) cfu/mL of test bacteria and incubated for 18 h at 37°C. Plates were screened for growth by spectrometry at 600 nm. Control wells did not receive any drugs. The FICI was calculated by the following equation: FICI= (MIC of agent A in combination/MIC of agent A alone) + (MIC of agent B in combination/MIC of agent B alone) (Odds, 2003). Synergy was defined as FICI ≤0.5, antagonism as FICI ≥4 and no interaction as 0.5 < FICI < 4. All FICI assays were carried out three times on three different days. FICIs were calculated as the mean values from three independent experiments.

### *In Vitro* Time–Kill Curves

Time–kill experiments were conducted to further characterize the synergistic activity of the nitrofurantoin and amikacin combination as previously described (Dong et al., 2017). In brief, an initial inoculum of ~10^4^ cfu/larva logarithmic-phase cells were incubated with amikacin in the presence and absence of nitrofurantoin and time–kill curves were compared to assess efficacy. Serial samples were obtained at 0, 3, 6, 9, and 24 h after incubation at 37°C. Bacterial counts were determined based on the quantitative cultures on MHA plates. Synergy was defined as achieving a ≥ 2 log_10_ cfu/mL reduction in bacterial growth at 24 h with the combination compared with the most active individual drug concentration used on its own (Gomara and Ramon-Garcia, 2019). Three independent experimental runs were performed.

### Antibiotic Resistance Evolution Under Nitrofurantoin and Amikacin Single or Combination Stress

After time–kill experiments, five clones used for viable count enumeration in 24 h were randomly selected for each experimental group from MHA plates. MIC values were measured for these clones to compare whether drug resistance developed under nitrofurantoin and amikacin selection pressure when used alone and in combination.

### Galleria mellonella Infection Model

A well-characterized *G. mellonella* model was used in this study as previously described publication (Dong et al., 2017). The *G. mellonella* larvae were obtained from Kaide Ruixin (Tianjin, China). The optimal infection doses of the study test strains were determined using *G. mellonella* larvae that were randomly distributed into six experimental groups (n=10/group or ~250 mg). These were then infected by injection of 10 μL of logarithmic phase *E. coli* cells (~10^4^ cfu/larva) into the last left proleg. After injection, the larvae were incubated in plastic Petri dishes at 37°C for 72 h and scored for survival daily. In all experiments, PBS injections were used as negative controls.

The *in vivo* efficacy of nitrofurantoin and amikacin alone and in combination were assessed in the same *G. mellonella* model caused by our study *E. coli* strains using the optimal infection doses as determined above (~10^4^ cfu/larva). At 2 h post-infection, animals were randomized to receive no therapy or nitrofurantoin and amikacin alone, and in combination (n = 10/group) (Seed and Dennis, 2008; Ahmad et al., 2010; Dong et al., 2017). The antibiotics were administered only once (10 μL) into the last right proleg with nitrofurantoin at 3.75 mg/kg, amikacin at 7.5 mg/kg alone or in combination at half doses (Beaucaire et al., 1991; Amabile-Cuevas and Arredondo-Garcia, 2011). Larvae were observed daily for 3 days and percent of larvae survival was calculated for each group (**Figure 3A**).

### Statistical Analysis

Bacterial counts were transformed to log_10_ values and the data were analyzed using Graphpad Prism 7.0 (GraphPad Software, San Diego, CA, USA). P values were determined using a two-sided, Mann–Whitney U-test. A P-value of ≤ 0.05 was considered significant. All data were presented as means ± SD.

## Results

### *In Vitro* Susceptibility and Interaction Assessment

**Figure 1:**
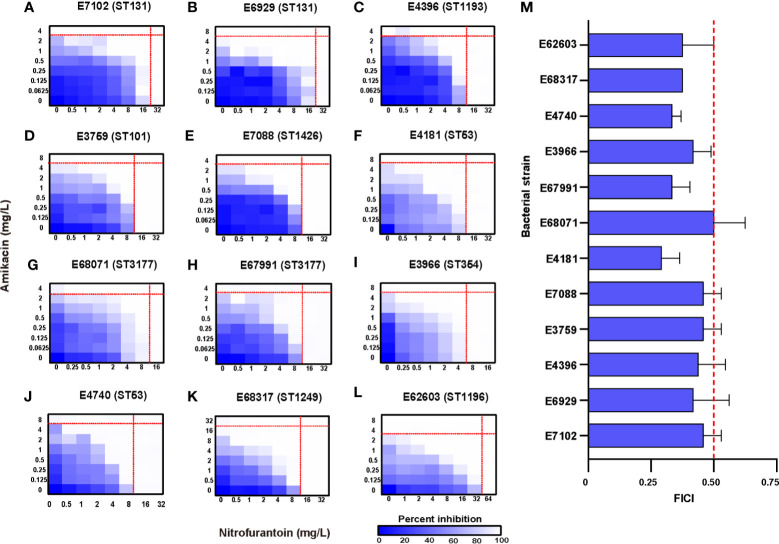
Potentiation of amikacin partnered with nitrofurantoin against 12 test strains. **(A–L)** Microdilution chequerboard assays are shown as 8×8 matrix heat map graphs. The blue colour gradient represents the bacterial cell density estimated by OD_600_. AMK, amikacin; NIF, nitrofurantoin. **(M)** FICI of the test strains where synergy is defined as a FIC index of ≤ 0.5. The thin red line represents the (MICs) for antibiotics used separately and the think one represents the FIC index of 0.5.

The MICs of 11 antibiotics were determined against our collection of clinical isolates. The MICs for amikacin ranged from 2 to 16 mg/L and all strains were susceptible. The MICs for nitrofurantoin ranged from 8 to 32 mg/L and all 12 UPEC strains were susceptible. These 12 clinical UPEC strains were classified as MDR *E. coli* (**Table 1** and **Table S1**). *In vitro* testing of amikacin/nitrofurantoin combinations indicated a synergistic action against all 12 UPEC strains with FICI values ranging from 0.292 ± 0.072 to 0.500 ± 0.125 (**Figure 1**). These data indicated that combination of amikacin and nitrofurantoin can synergize to combat MDR UPEC strains.

### *In Vitro* Time–Kill Curves

**Figure 2:**
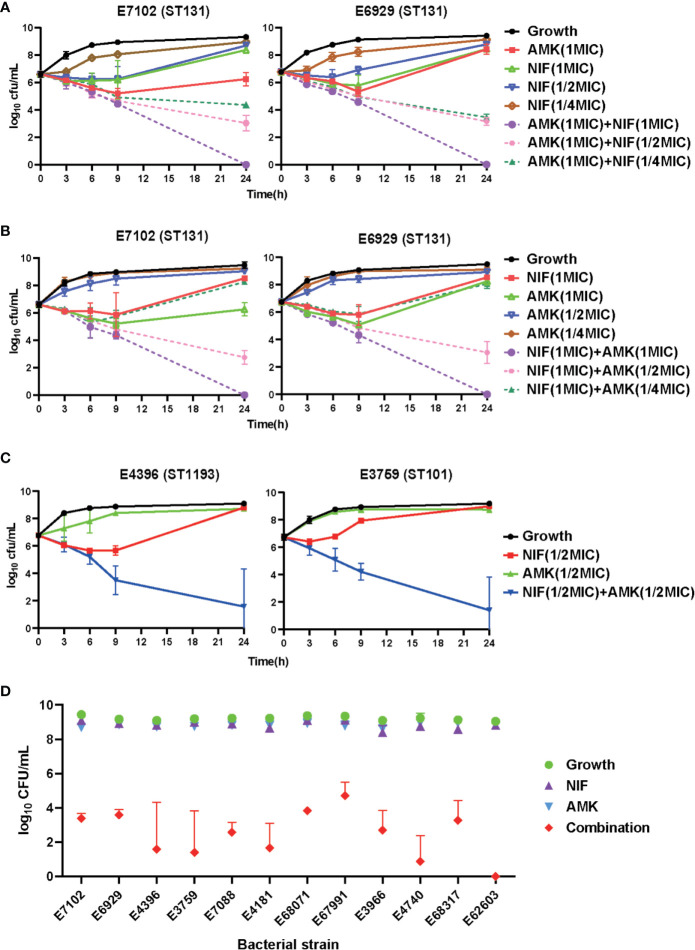
*In vitro* time–kill curves using amikacin and nitrofurantoin alone and in combination against the indicated test strains. **(A–C)** Combinatorial bactericidal activity of amikacin and nitrofurantoin against ST131 UPEC strains. Mean ± standard error from three independent experiments are shown. AMK, amikacin; NIF, nitrofurantoin. **(D)** Growth after 24 h for all the test strains using amikacin and nitrofurantoin alone and in combinations compared to the control.

We then performed kinetic time–kill assays for all test strains to better evaluate the pharmacodynamics of the amikacin and nitrofurantoin interaction. We examined time–kill curves representing log_10_ changes in bacterial burden using the ST131 UPEC strains E7102 and E6929 over 24 h following exposure to amikacin (1×MIC) in the presence of increasing amikacin concentrations (1/4−1×MIC). The addition of nitrofurantoin to amikacin increased *in vitro* bactericidal activity compared with nitrofurantoin alone. Similarly, the addition of amikacin to nitrofurantoin also significantly increased *in vitro* bactericidal activity (**Figures 2A, B**). We then tested amikacin at 1/2 MIC alone and in combination with 1/2 MIC nitrofurantoin, to observe whether they could have a good bactericidal effect under the sub-inhibition concentration. The combination therapy resulted in synergistic effects against all 12 clinical UPEC strains. For instance, the combination therapy caused more than a 2 log_10_ cfu/mL reduction for all 12 UPEC strains as compared to the most active antibiotic alone (**Figure 2C** and **Figure S1**). The amikacin/nitrofurantoin combination significantly increased *in vitro* antimicrobial activity and resulted in a rapid killing of the bacterial test strains for 9 combination groups caused reductions as compared with the most active antibiotic alone that ranged from 4.055 ± 1.050 to 8.714 ± 0.131 cfu/mL (**Table S2**). The combination group against *E. coli* strain E67991 was obtained most weakly synergistic effects, but also caused more than 4 log10 cfu/mL reductions and showing bactericidal action. Against *E. coli* strain E4740 and E62603, the combination group almost completely elimination the bacteria at 24 h of incubation, and the bacterial burden less than 1 log_10_ cfu/mL (**Figure 2D**).

### Antibiotic Resistance Evolution Under Nitrofurantoin and Amikacin Single or Combination Stress

Considering the pronounced effects that the combination therapy had on *in vitro* bacterial growth, five clones were selected for MIC determinations. In the 12 nitrofurantoin groups, 3 MICs were increased while the other 9 were unchanged. In contrast, 5 amikacin MICs were decreased, 5 were unchanged and 2 groups displayed MIC increases. The 12 amikacin groups displayed 10 MICs that were increased and 2 unchanged while 4 nitrofurantoin MICs were decreased and 8 remained unchanged. When the amikacin groups were compared with the combination group, the amikacin MICs were decreased in 10/12 of the combination groups and in the other two groups, one group was unchanged and one increased. Compared to the nitrofurantoin groups, the nitrofurantoin MICs had decreased in 3/12 of the combination groups while the remaining 9 groups were unchanged (**Figure S2**).

### *In Vivo* Synergistic Efficacy

**Figure 3:**
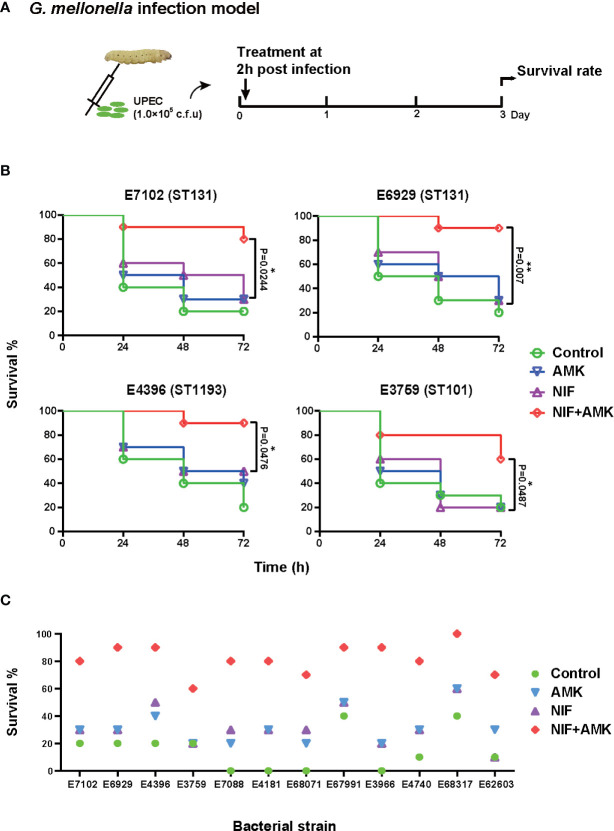
Therapeutic effects of amikacin combined with nitrofurantoin in the *G. mellonella* model. **(A)** Scheme of the experimental protocol for the *G. mellonella* model. **(B)** Survival rates of amikacin and nitrofurantoin alone (most effective) and in combination treatment in an experimental *G. mellonella* model caused by the indicated UPEC strains. **(C)** Survival rates after 72 h caused by the indicated UPEC strains. (∗) p < 0.05 and (∗∗) p < 0.01.

The amikacin/nitrofurantoin combination showed significant synergistic effects *in vitro* so we investigated whether these effects in the *in vivo G. mellonella* model using amikacin and nitrofurantoin at half clinical dosages. The amikacin and nitrofurantoin monotherapies were ineffective against our two ST131 UPEC strains (E7102 and E6929) but the combination therapy resulted in 80–90% survival after 72 h. The combination therapy also significantly increased survival from infections with the E4396 and E3759 strains (**Figure 3B**). The use of amikacin monotherapy significantly increased *G. mellonella* survival from infections in only a single UPEC strain (E4181). Similarly, nitrofurantoin monotherapy significantly increased *G. mellonella* survival from infections caused by two strains; E4181 and E68071. Therefore overall, amikacin or nitrofurantoin given at subinhibitory concentrations was not effective in the treatment of UPEC infections in the *G. mellonella* model. In contrast, the amikacin/nitrofurantoin combination significantly increased survival from infections caused by all 12 study UPEC strains. More importantly, the combination therapy significantly increased survival compared with amikacin or nitrofurantoin monotherapies with 11/12 of the test strains (p < 0.05). Only one UPEC strain E68071 generated a non-significant P value (0.0957). Overall, we found increases in survival with the combination therapies that increased survival against challenge by our UPEC test strains from 40 to 70% (**Figure 3C** and **Figure S3**).

## Discussion

Almost all patients with UTI are treated with antibiotics that generates annual costs estimated for the United States at $2.14 billion (Brown et al., 2005). The antimicrobial agents most commonly used to treat uncomplicated UTI include the combination trimethoprim and sulfamethoxazole, trimethoprim, β-lactams, fluoroquinolones, nitrofurantoin, and fosfomycin, third-generation cephalosporins, aminoglycosides and carbapenems (Jancel and Dudas, 2002; Koningstein et al., 2014) and this wide range of treatment options belies the serious threat that these MDR organisms pose. The increasing prevalence of antibiotic-resistant uropathogens has begun to limit the effectiveness of our existing antibiotic arsenal (Barber et al., 2013). Combinations of antibiotics are commonly used in medicine to broaden the antimicrobial spectrum and generate synergistic effects and this therapy has proven effective against MDR bacteria (Gomara and Ramon-Garcia, 2019). For example, the use of oral cephalosporin and β-lactamase inhibitor combinations for ESBL-producing Enterobacteriaceae UTI (Stewart et al., 2020).

Nitrofurantoin and amikacin are both used for the treatment and prevention of UTIs. To the best of our knowledge, we are the first to report of the synergistic effect of amikacin and nitrofurantoin against UPEC in the *G. mellonella* model. This provides a new strategy for the treatment of UTIs caused by UPEC. It is worth noting that aminoglycoside drugs (such as gentamicin and tobramycin) previously been reported can improve the sensitivity of UPEC to Nitrofurantoin after treatment (Adamus-Bialek et al., 2019). One challenge presented by drug combinations is the requirement to determine coincident pharmacological properties such as tissue distribution and penetration (Ejim et al., 2011). The nitrofurantoin/amikacin combination can avoid this problem because these two antibiotics are in current used for UTI treatment and prevention. In general, maximum urine concentrations of nitrofurantoin vary from 15 mg/L to 230 mg/L and were found between ~ 3 and 10 h after dosing, depending on the crystal size, formulation of the nitrofurantoin product and the fasting status of the subject (Wijma et al., 2018). Conventional amikacin is almost entirely excreted unchanged in the urine within hours after administration in all species studied (Fielding et al., 1999).

The synergistic mechanism of nitrofurantoin and amikacin is not clear because nitrofurantoin possesses several mechanisms of antimicrobial action that involve damage to DNA and ribosomes (Woody-Karrer and Greenberg, 1963; Jenkins and Bennett, 1976; Huttner et al., 2015). Amikacin targets the bacterial ribosome and inhibits translation by causing misreading and hindering translocation (Taber et al., 1987; Allison et al., 2011). Both these drugs target the ribosome and this is the most likely site of action for the combination. In addition, nitrofurantoin stimulates the production of reactive oxygen species (ROS) (Garcia Martinez et al., 1995) that can facilitate the entry of aminoglycosides and subsequent bacterial killing (Ezraty et al., 2013). Furthermore, aminoglycosides have collateral sensitivity with many antibiotics, nitrofurantoin has also been reported to be collateral sensitivity with tigecycline, mecillinam and protamine, therefore, whether nitrofurantoin has synergistic sensitivity with amikacin is also very much studied (Suzuki et al., 2014; Pal et al., 2015; Roemhild et al., 2020).

The results of the time-kill assays in this study demonstrated that nitrofurantoin/amikacin at 1/2 MIC concentration displayed synergistic bactericidal effects and indicated that combination therapy can reduce antibiotic dosage. In addition, at half of the clinically recommended dose, the combined treatment group significantly increased the survival rate of larva compared with the single treatment group. After 24 h under nitrofurantoin or amikacin stress, the MIC values of the corresponding drugs increased to different degrees, especially in the case of amikacin. However, the MIC values for both nitrofurantoin and amikacin were decreased in the combination group compared with the single drug group, although the decrease was less than 2-fold. This suggests drug combination strategies can be effective against MDR bacteria while slowing down the development of antibiotic resistance.

In the *G. mellonella* infection model, the combination treatment significantly improved larvae survival compared with the most active antibiotic alone. These results were consistent with the *in vitro* time kill assays except for a single UPEC strain, 68071. These results indicated that the *G. mellonella* model is useful for assessing the *in vivo* efficacy of anti-UPEC agents. Previous studies have indicated that *in vivo* synergism results were not always directly related to the *in vitro* results (Thieme et al., 2020). This may be due to strain differences and the test antibiotics. The *G. mellonella* model enables a rapid, economical and reproducible model to assess the synergistic effects of antimicrobials in an *in vivo* setting (Cools et al., 2019).

There are several limitations to this study that should be noted. For example, we did not have a full complement of strains with low level resistance to both nitrofurantoin and amikacin. In addition, we only tested UPEC strains in the *G. mellonella* model rather than the murine urinary tract infection model and future will address this concern. There was a certain gap in the model construction compare with the natural infection, and we did not take into account factors such as the formation of biofilm by UPEC in animal, these require further study. Moreover, based on our current findings, further investigations are necessary to examine the effectiveness of this combination in PK/PD models to optimize the dose regimen. The tests of synergy for this drug combination must also be linked to patient outcome (Doern, 2014).

In summary, we confirmed that the combination of nitrofurantoin and amikacin possesses a significantly synergistic effect on MDR UPEC *in vitro*. In addition, we demonstrated for the first time that this drug combination was significantly synergistic effect on MDR UPEC in the *G. mellonella* model. Our findings constitute an alternative and promising therapeutic option for the treatment of UTIs caused by MDR UPEC.

## Data Availability Statement

The datasets presented in this study can be found in online repositories. The names of the repository/repositories and accession number(s) can be found below: NCBI BioProject [accession: PRJNA678682].

## Ethics Statement

Clinical strains isolated from humans in this study were provided by the Third Affiliated Hospital of Sun Yat-sen University. This study was carried out in accordance with the recommendations of ethical guidelines of South China Agricultural University. SCAU Institutional Ethics Committee did not require the study to be reviewed or approved by an ethics committee because we are not involved in the isolation of bacteria.

## Author Contributions

Z-XZ and Z-HC contributed equally in this study. JS, Y-HL, and X-PL designed the study. Z-HC, Z-XZ, TT, X-JL, Z-JZ, and W-NN carried out the experiments. JS, Z-HC, YY, and Y-FZ analyzed the data. JS, Z-HC, L-XF, and Z-XZ wrote the draft of the manuscript. All authors contributed to the article and approved the submitted version.

## Funding

This work was supported by National Natural Science Foundation of China (31972735), the National Key Research and Development Program of China (2016YFD0501300), the Program for Innovative Research Team in the University of Ministry of Education of China (IRT_17R39), and the 111 Project (D20008).

## Conflict of Interest

The authors declare that the research was conducted in the absence of any commercial or financial relationships that could be construed as a potential conflict of interest.

## References

[B1] Abbasi H. Ranjbar R. (2018). The prevalence of quinolone resistance genes of A, B, S in Escherichia coli strains isolated from three major hospitals in Tehran, Iran. Cent. Eur. J. Urol. 71, 129–133. 10.5173/ceju.2018.1539 PMC5926638 29732219

[B2] Adamus-Bialek W. Wawszczak M. Arabski M. Majchrzak M. Gulba M. Jarych D. (2019). Ciprofloxacin, amoxicillin, and aminoglycosides stimulate genetic and phenotypic changes in uropathogenic Escherichia coli strains. Virulence 10, 260–276. 10.1080/21505594.2019.1596507 30938219 PMC6527016

[B3] Ahmad S. Hunter L. Qin A. Mann J. B. van Hoek M. Qin A. (2010). Azithromycin effectiveness against intracellular infections of Francisella. BMC Microbiol. 10, 123. 10.1186/1471-2180-10-123 20416090 PMC2881020

[B4] Allison K. R. Brynildsen M. P. Collins J. J. (2011). Metabolite-enabled eradication of bacterial persisters by aminoglycosides. Nature 473, 216–220. 10.1038/nature10069 21562562 PMC3145328

[B5] Amabile-Cuevas C. F. Arredondo-Garcia J. L. (2011). Antimicrobial activity data in support of nitrofurantoin three times per day. J. Antimicrob. Chemother. 66, 1652–1653. 10.1093/jac/dkr170 21525023

[B6] Ayukekbong J. A. Ntemgwa M. Atabe A. N. (2017). The threat of antimicrobial resistance in developing countries: causes and control strategies. Antimicrob Resist. Infect. Control 6, 47. 10.1186/s13756-017-0208-x 28515903 PMC5433038

[B7] Bankevich A. Nurk S. Antipov D. Gurevich A. A. Dvorkin M. Kulikov A. S. (2012). SPAdes: a new genome assembly algorithm and its applications to single-cell sequencing. J. Comput. Biol. 19, 455–477. 10.1089/cmb.2012.0021 22506599 PMC3342519

[B8] Barber A. E. Norton J. P. Spivak A. M. Mulvey M. A. (2013). Urinary tract infections: current and emerging management strategies. Clin. Infect. Dis. 57, 719–724. 10.1093/cid/cit284 23645845 PMC3739462

[B9] Beaucaire G. Leroy O. Beuscart C. Karp P. Chidiac C. Caillaux M. (1991). Clinical and bacteriological efficacy, and practical aspects of amikacin given once daily for severe infections. J. Antimicrob. Chemother. 27 (Suppl C), 91–103. 10.1093/jac/27.suppl_C.91 1856149

[B10] Brochado A. R. Telzerow A. Bobonis J. Mateus M. Selkrig J. (2018). Species-specific activity of antibacterial drug combinations. Nature 57, 259–263. 10.1038/s41586-018-0278-9 PMC6219701 29973719

[B11] Brown P. Ki M. Foxman B. (2005). Acute pyelonephritis among adults - Cost of illness and considerations for the economic evaluation of therapy. Pharmacoeconomics 23, 1123–1142. 10.2165/00019053-200523110-00005 16277548

[B12] Cools F. Torfs E. Aizawa J. Vanhoutte B. Maes L. Caljon G. (2019). Optimization and Characterization of a Galleria mellonella Larval Infection Model for Virulence Studies and the Evaluation of Therapeutics Against Streptococcus pneumoniae. Front. Microbiol. 10, 311. 10.3389/fmicb.2019.00311 30846978 PMC6394149

[B13] Daoud Z. Salem Sokhn E. Masri K. Matar G. M. Doron S. (2015). Escherichia coli Isolated from Urinary Tract Infections of Lebanese Patients between 2005 and 2012: Epidemiology and Profiles of Resistance. Front. Med. 2:26. 10.3389/fmed.2015.00026 PMC4415468 25984513

[B14] Doern C. D. (2014). When does 2 plus 2 equal 5? A review of antimicrobial synergy testing. J. Clin. Microbiol. 52, 4124–4128. 10.1128/JCM.01121-14 24920779 PMC4313275

[B15] Dong C. L. Li L. X. Cui Z. H. Chen S. W. Xiong Y. Q. Lu J. Q. (2017). Synergistic Effect of Pleuromutilins with Other Antimicrobial Agents against Staphylococcus aureus In Vitro and in an Experimental Galleria mellonella Model. Front. Pharmacol. 8, 553. 10.3389/fphar.2017.00553 28874907 PMC5572081

[B16] Ejim L. Farha M. A. Falconer S. B. Wildenhain J. Coombes B. K. Tyers M. (2011). Combinations of antibiotics and nonantibiotic drugs enhance antimicrobial efficacy. Nat. Chem. Biol. 7, 348–350. 10.1038/nchembio.559 21516114

[B17] Ezraty B. Vergnes A. Banzhaf M. Duverger Y. Huguenot A. Brochado A. R. (2013). Fe-S cluster biosynthesis controls uptake of aminoglycosides in a ROS-less death pathway. Science 340, 1583–1587. 10.1126/science.1238328 23812717

[B18] Farajzadah Sheikh A. Goodarzi H. Yadyad M. J. Aslani S. Amin M. Jomehzadeh N. (2019). Virulence-associated genes and drug susceptibility patterns of uropathogenic Escherichia coli isolated from patients with urinary tract infection. Infect. Drug Resist. 12, 2039–2047. 10.2147/IDR.S199764 31410031 PMC6646852

[B19] Fielding R. M. Moon-McDermott L. Lewis R. O. Horner M. (1999). Pharmacokinetics and urinary excretion of amikacin in low-clearance unilamellar liposomes after a single or repeated intravenous administration in the rhesus monkey. Antimicrob Agents Chemother. 43, 503–509. 10.1128/AAC.43.3.503 10049258 PMC89151

[B20] Garcia Martinez P. Winston G. W. Metash-Dickey C. O'Hara S. C. Livingstone D. R. (1995). Nitrofurantoin-stimulated reactive oxygen species production and genotoxicity in digestive gland microsomes and cytosol of the common mussel (Mytilus edulis L.). Toxicol. Appl. Pharmacol. 131, 332–341. 10.1006/taap.1995.1076 7716774

[B21] Godaly G. Ambite I. Svanborg C. (2015). Innate immunity and genetic determinants of urinary tract infection susceptibility. Curr. Opin. Infect. Dis. 28, 88–96. 10.1097/QCO.0000000000000127 25539411 PMC4286230

[B22] Gomara M. Ramon-Garcia S. (2019). The FICI paradigm: Correcting flaws in antimicrobial in vitro synergy screens at their inception. Biochem. Pharmacol. 163, 299–307. 10.1016/j.bcp.2019.03.001 30836058

[B23] Goodlet K. J. Benhalima F. Z. Nailor M. D. (2018). A Systematic Review of Single-Dose Aminoglycoside Therapy for Urinary Tract Infection: Is It Time To Resurrect an Old Strategy? Antimicrob Agents Chemother. 63, 02165-18. 10.1128/AAC.0216518 PMC6325212 30397061

[B24] Gupta K. Hooton T. M. Naber K. G. Björn Wullt B. Colgan R. Miller L. G. (2011). International clinical practice guidelines for the treatment of acute uncomplicated cystitis and pyelonephritis in women: A 2010 update by the Infectious Diseases Society of America and the European Society for Microbiology and Infectious Diseases. Clin. Infect. Dis. 52, e103–e120. 10.1093/cid/cir102 21292654

[B25] Halaji M. Feizi A. Mirzaei A. Saraie H. Fayyazi A. Ashraf A. (2020a). The Global Prevalence of Class 1 Integron and Associated Antibiotic Resistance in Escherichia coli from Patients with Urinary Tract Infections, a Systematic Review and Meta-Analysis. Microb. Drug Resist. 26, 1208–1218. 10.1089/mdr.2019.0467 32282274

[B26] Halaji M. Shahidi S. Atapour A. Ataei B. Feizi A. Havaei S. A. (2020b). Characterization of Extended-Spectrum beta-Lactamase-Producing Uropathogenic Escherichia coli Among Iranian Kidney Transplant Patients. Infect. Drug Resist. 13, 1429–1437. 10.2147/IDR.S248572 32523361 PMC7237106

[B27] Han S. B. Lee S. C. Lee S. Y. Jeong D. C. Kang J. H. (2015). Aminoglycoside therapy for childhood urinary tract infection due to extended-spectrum beta-lactamase-producing Escherichia coli or Klebsiella pneumoniae. BMC Infect. Dis. 15, 414. 10.1186/s12879-015-1153-z 26464143 PMC4604622

[B28] Huttner A. Verhaegh E. M. Harbarth S. Muller A. E. Theuretzbacher U. Mouton J. W. (2015). Nitrofurantoin revisited: a systematic review and meta-analysis of controlled trials. J. Antimicrob. Chemother. 70, 2456–2464. 10.1093/jac/dkv147 26066581

[B29] Ingalsbe M. L. Wojciechowski A. L. Smith K. A. Mergenhagen K. A. (2015). Effectiveness and safety of nitrofurantoin in outpatient male veterans. Ther. Adv. Urol. 7, 186–193. 10.1177/1756287215581556 26445598 PMC4580093

[B30] Jancel T. Dudas V. (2002). Management of uncomplicated urinary tract infections. West. J. Med. 176, 51–55. 10.1136/ewjm.176.1.51 11788540 PMC1071654

[B31] Jenkins S. T. Bennett P. M. (1976). Effect of mutations in deoxyribonucleic acid repair pathways on the sensitivity of Escherichia coli K-12 strains to nitrofurantoin. J. Bacteriol. 125, 1214–1216. 10.1128/JB.125.3.1214-1216.1976 767322 PMC236203

[B32] Johnson J. R. Stamm W. E. (1987). Diagnosis and treatment of acute urinary tract infections. Infect. Dis. Clin. North Am. 1, 773–791. 10.1097/00000441-198912000-00009 3333658

[B33] Klein R. D. Hultgren S. J. (2020). Urinary tract infections: microbial pathogenesis, host–pathogen interactions and new treatment strategies. Nat. Rev. Microbiol. 18, 211–226. 10.1038/s41579-020-0324-0 32071440 PMC7942789

[B34] Koningstein M. van der Bij A. K. de Kraker M. E. Monen J. C. Muilwijk J. de Greeff S. C. (2014). Recommendations for the empirical treatment of complicated urinary tract infections using surveillance data on antimicrobial resistance in the Netherlands. PLoS One 9, e86634. 10.1371/journal.pone.0086634 24489755 PMC3904917

[B35] Leibovici L. Vidal L. Paul M. (2009). Aminoglycoside drugs in clinical practice: an evidence-based approach. J. Antimicrob. Chemother. 63, 246–251. 10.1093/jac/dkn469 19022778

[B36] Muller A. E. Verhaegh E. M. Harbarth S. Mouton J. W. Huttner A. (2017). Nitrofurantoin’s efficacy and safety as prophylaxis for urinary tract infections: a systematic review of the literature and meta-analysis of controlled trials. Clin. Microbiol. Infect. 23, 355–362. 10.1016/j.cmi.2016.08.003 27542332

[B37] Odds F. C. (2003). Synergy, antagonism, and what the chequerboard puts between them. J. Antimicrob. Chemother. 52, 1. 10.1093/jac/dkg301 12805255

[B38] Pal C. Papp B. Lazar V. (2015). Collateral sensitivity of antibiotic-resistant microbes. Trends Microbiol. 23, 401–407. 10.1016/j.tim.2015.02.009 25818802 PMC5958998

[B39] Phan M. D. Bottomley A. L. Peters K. M. Harry E. J. Schembri M. A. (2020). Uncovering novel susceptibility targets to enhance the efficacy of third-generation cephalosporins against ESBL-producing uropathogenic Escherichia coli. J. Antimicrob. Chemother. 75, 1415–1423. 10.1093/jac/dkaa023 32073605

[B40] Raeispour M. Ranjbar R. (2018). Antibiotic resistance, virulence factors and genotyping of Uropathogenic Escherichia coli strains. Antimicrob. Resist. Infect. Control 7, 118. 10.1186/s13756-018-0411-4 30305891 PMC6171155

[B41] Roemhild R. Linkevicius M. Andersson D. I. (2020). Molecular mechanisms of collateral sensitivity to the antibiotic nitrofurantoin. PLoS Biol. 18, e3000612. 10.1371/journal.pbio.3000612 31986134 PMC7004380

[B42] Rohde A. M. Wiese-Posselt M. Zweigner J. Schwab F. Mischnik A. Seifert H. (2018). High admission prevalence of fluoroquinolone resistance in third-generation cephalosporin-resistant Enterobacteriaceae in German university hospitals. J. Antimicrob. Chemother. 73, 1688–1691. 10.1093/jac/dky040 29490046

[B43] Seed K. D. Dennis J. J. (2008). Development of Galleria mellonella as an alternative infection model for the Burkholderia cepacia complex. Infect. Immun. 76, 1267–1275. 10.1128/IAI.01249-07 18195031 PMC2258804

[B44] Soto S. M. Jimenez de Anta M. T. Vila J. (2006). Quinolones induce partial or total loss of pathogenicity islands in uropathogenic Escherichia coli by SOS-dependent or -independent pathways, respectively. Antimicrob. Agents Chemother. 50, 649–653. 10.1128/AAC.50.2.649-653.2006 16436722 PMC1366871

[B45] Spring P. J. Sharpe D. M. Hayes M. W. (2001). Nitrofurantoin and peripheral neuropathy: a forgotten problem? Med. J. Aust. 174, 153–154. 10.5694/j.1326-5377.2001.tb143200.x 11247627

[B46] Stewart A. G. Harris P. N. A. Henderson A. Schembri M. A. Paterson D. L. (2020). Oral cephalosporin and beta-lactamase inhibitor combinations for ESBL-producing Enterobacteriaceae urinary tract infections. J. Antimicrob. Chemother. 75, 2384–2393. 10.1093/jac/dkaa183 32443141

[B47] Suzuki S. Horinouchi T. Furusawa C. (2014). Prediction of antibiotic resistance by gene expression profiles. Nat. Commun. 5, 5792. 10.1038/ncomms6792 25517437 PMC4351646

[B48] Taber H. W. Mueller J. P. Miller P. F. Arrow A. S. (1987). Bacterial uptake of aminoglycoside antibiotics. Microbiol. Rev. 51, 439–457. 10.1128/MMBR.51.4.439-457.1987 3325794 PMC373126

[B49] Tchesnokova V. Riddell K. Scholes D. Johnson J. R. Sokurenko E. V. (2019). The Uropathogenic Escherichia coli Subclone Sequence Type 131-H30 Is Responsible for Most Antibiotic Prescription Errors at an Urgent Care Clinic. Clin. Infect. Dis. 68, 781–787. 10.1093/cid/ciy523 29961840 PMC6376094

[B50] Thieme L. Hartung A. Makarewicz O. Pletz M. W. (2020). In vivo synergism of ampicillin, gentamicin, ceftaroline and ceftriaxone against Enterococcus faecalis assessed in the Galleria mellonella infection model. J. Antimicrob. Chemother. 75, 2173–2181. 10.1093/jac/dkaa129 32357212

[B51] White R. L. Burgess D. S. Manduru M. Bosso J. A. (1996). Comparison of three different in vitro methods of detecting synergy: time-kill, checkerboard, and E test. Antimicrob Agents Chemother. 40, 1914–1918. 10.1128/AAC.40.8.1914 8843303 PMC163439

[B52] Wijma R. A. Huttner A. Koch B. C. P. Mouton J. W. Muller A. E. (2018). Review of the pharmacokinetic properties of nitrofurantoin and nitroxoline. J. Antimicrob. Chemother. 73, 2916–2926. 10.1093/jac/dky255 30184207

[B53] Woody-Karrer P. Greenberg J. (1963). Resistance and Cross Resistance of Escherichia Coli S Mutants to the Radiomimetic Agent Nitrofurazone. J. Bacteriol. 85, 1208–1216. 10.1128/JB.85.6.1208-1216.1963 14047210 PMC278321

[B54] Yeh P. Tschumi A. I. Kishony R. (2006). Functional classification of drugs by properties of their pairwise interactions. Nat. Genet. 38, 489–494. 10.1038/ng1755 16550172

