# Supplementary material for: Nitrofurantoin Combined With Amikacin: A Promising Alternative Strategy for Combating MDR Uropathogenic Escherichia coli
Source: Front Cell Infect Microbiol. 2020 Dec 21;10:608547. doi: 10.3389/fcimb.2020.608547 (PMC7779487; doi:10.3389/fcimb.2020.608547)
Supplement: Supplementary file 1 [file DataSheet_1.pdf]

Supplementary Information for

Title: Nitrofurantoin combined with amikacin: A promising alternative strategy for treating urinary tract infections caused by MDR Enterobacteriaceae

Zi-Xing Zhong[1,3#], Ze-Hua Cui[1,3#], Xiao-Jie Li[4], Tian Tang[1,3], Zi-Jian Zheng[1,3], Wei-Na Ni[1,3], Liang-Xing Fang[1,3], Yu-Feng Zhou[1,3], Yang Yu[1,3], Ya-Hong Liu[1,2,3], Xiao-Ping Liao[1,2,3], Jian Sun[1,2,3*].

[1] National Risk Assessment Laboratory for Antimicrobial Resistance of Animal Original Bacteria, South China Agricultural University, Guangzhou, China.

[2] Guangdong Laboratory for Lingnan Modern Agriculture, Guangzhou, 510642, China.

[3] Guangdong Provincial Key Laboratory of Veterinary Pharmaceutics Development and Safety Evaluation, South China Agricultural University, Guangzhou 510642, China.

[4] Department of Laboratory Medicine, The Third Affiliated Hospital, Sun Yat-sen University, Guangzhou 510630, China

[#]The authors contributed equally to this work: Zi-Xing Zhong and Ze-Hua Cui.

* Correspondence author:

Jian Sun.

E-mail: jiansun@scau.edu.cn.

Tel: +86-020-85285507; Fax: +86-020-85285507

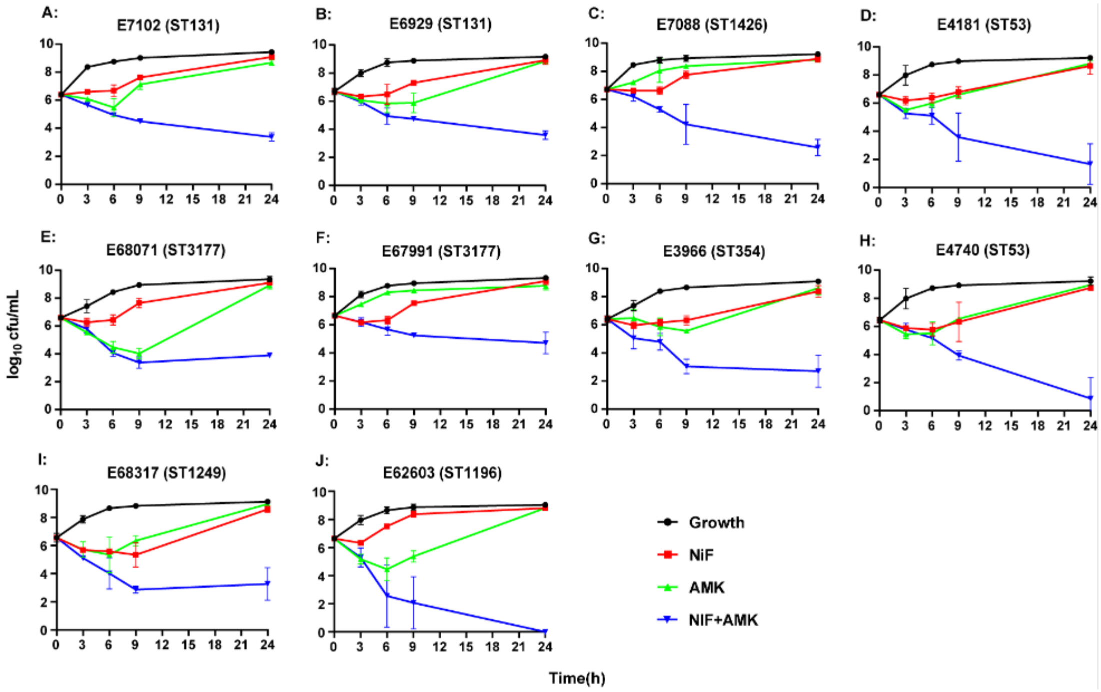

Figure S1. The results of time-kill curves of amikacin and nitrofurantoin alone and in combinations against 10 non-ST131 test UPEC strains.

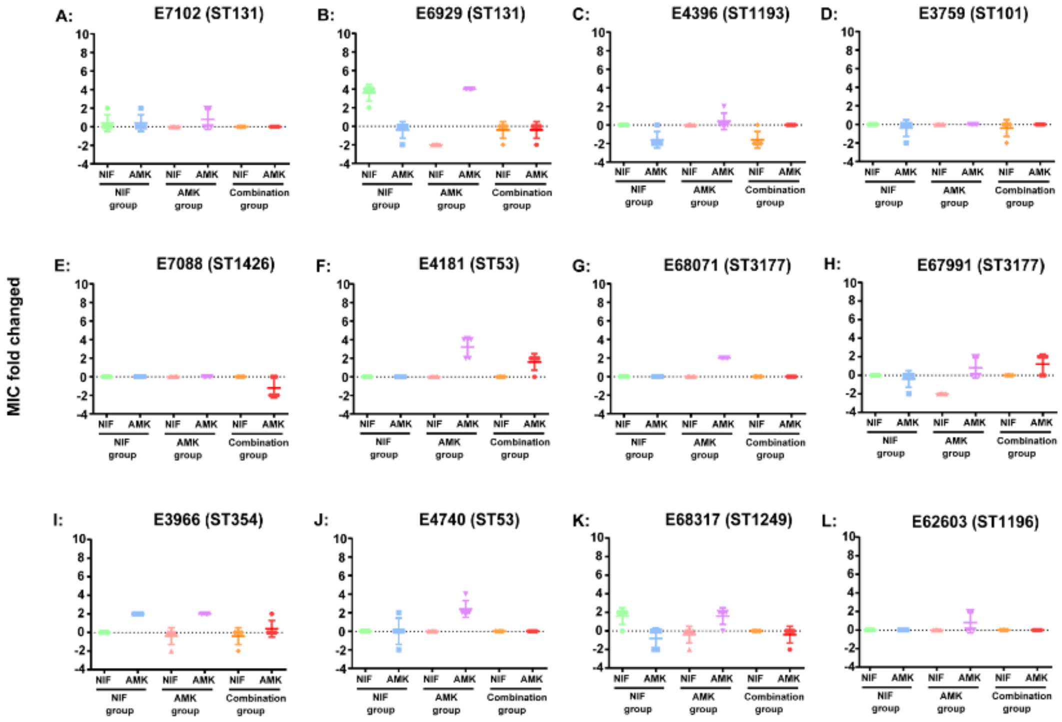

Figure S.2

MICs changed of AMK and NIF after 24 h under stress of drug single or combination.

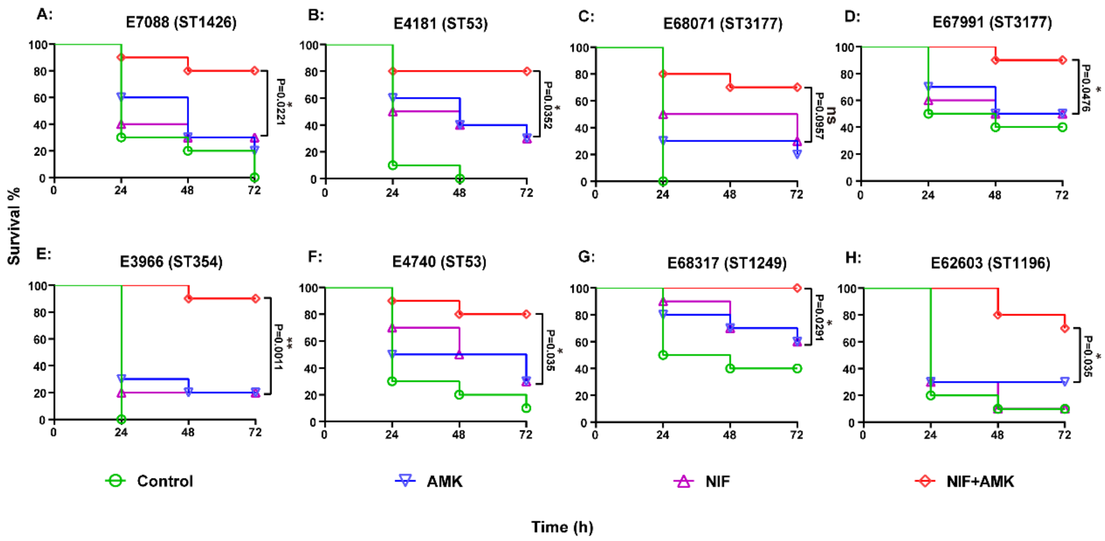

Figure S.3 Survival rates of amikacin and nitrofurantoin alone and in combination treatment in an experimental G. mellonella model caused by test 8 non-ST131 UPEC strains. (ns) No significant ,(∗) $p < 0.05$ and (∗∗) $p < 0.01$

Table S.1 *In vitro* antimicrobial susceptibility profiles for clinical strains.

E. coli strains	Relevant genotype	Resistant genes
ATCC25922	ST73; ATCC strain	NA
E7102	ST131	*aadA5*、*bla*$_{CTX-M-15}$、*erm*(B)、*mdf*(A)、*mph*(A)、*sul1*、*dfrA17*
E6929	ST131	*aadA5*、*bla*$_{CTX-M-15}$、*erm*(B)、*mdf*(A)、*sul1*、*dfrA17*
E4396	ST1193	*aadA5*、*aph(3'')-Ib*、*aph(6)-Id*、*bla*$_{CTX-M-27}$、*mdf*(A)、*mph*(A)、*sul1*、*sul2*、*tet*(A)、*dfrA17*
E3759	ST101	*aph(3'')-Ib*、*aph(6)-Id*、*bla*$_{CTX-M-55}$、*mdf*(A)、*sul2*、*tet*(A)、*dfrA14*
E7088	ST1426	*aph(3'')-Ib*、*aph(6)-Id*、*bla*$_{CTX-M-55}$、*mdf(A)*、*qnrS1*、*sul2*、*dfrA14*
E4181	ST53	*bla*$_{CTX-M-55}$、*bla*$_{TEM-1B}$、*mdf*(A)
E68071	ST3177	*bla*$_{CTX-M-15}$、*mdf*(A)、*tet*(B)
E67991	ST3177	*aac(3)-IId*、*aadA2*、*bla*$_{CTX-M-55}$、*bla*$_{TEM-1B}$、mdf(A)、*sul1*、*dfrA12*
E3966	ST354	*aac(3)-IId*、*aadA5*、*aph(3'')-Ib*、*aph(6)-Id*、*bla*$_{CTX-M-24}$、*bla*$_{TEM-1B}$、*mdf*(A)、*mph*(A)、*sul1*、*sul2*、*tet*(B)
E4740	ST53	*aac(3)-IId*、*aph(3'')-Ib*、*aph(6)-Id*、*bla*$_{CTX-M-55}$、*bla*$_{TEM-1B}$、*mdf*(A)、*mph*(A)、*sul2*、*tet*(A)
E68317	ST1249	*aac(6')-Ib3*、*aadA22* 、*bla*$_{CTX-M-14}$、*erm*、*tet*(A)
E62603	ST1196	*aac(3)-IId*、*aadA22*、*aph(3'')-Ib*、*aph(6)-Id*、*bla*$_{CTX-M-55}$、*LAP-2*、*bla*$_{TEM-1B}$、*lnu*(F)、*mdf*(A)、*mph*(A)、*catA2*、*qnrS1*、*ARR-2*、*sul2*、*sul3*、*tet*(A)、*dfrA14*

Table S.2 The log change (log_{10} cfu/mL) between the combinations vs. initial inoculum and the most active single agent after 24 h of incubation.

					Colony change（log_{10} cfu/mL) at 24 h	
Strain	Growth	NIF	AMK	Combination	vs. initial inoculum	vs. most active drug
E7102	9.434±0.078	9.084±0.008	8.681±0.203	3.380±0.301	-6.054±0.26	-5.397±0.523
E6929	9.163±0.177	8.910±0.301	8.849±0.149	3.589±0.299	-5.575±0.443	-5.221±0.154
E4396	9.093±0.024	8.806±0.113	8.716±0.163	1.583±2.741	-7.510±2.753	-7.060±2.776
E3759	9.188±0.048	8.990±0.117	8.747±0.203	1.401±2.427	-7.786±2.474	-7.346±2.462
E7088	9.222±0.069	8.884±0.245	8.817±0.094	2.577±0.584	-6.645±0.554	-6.202±0.432
E4181	9.221±0.151	8.649±0.600	8.814±0.216	1.661±1.443	-7.560±1.586	-6.829±1.577
E68071	9.361±0.209	9.101±0.051	8.921±0.257	3.822±0.122	-5.539±0.196	-5.058±0.126
E67991	9.339±0.124	9.113±0.247	8.771±0.282	4.716±0.768	-4.623±0.884	-4.055±1.050
E3966	9.086±0.126	8.383±0.416	8.616±0.478	2.695±1.142	-6.391±1.017	-5.688±0.815
E4740	9.220±0.300	8.747±0.203	8.954±0.222	0.867±1.502	-8.352±1.213	-7.852±1.514
E68317	9.121±0.163	8.577±0.215	8.956±0.049	3.269±1.155	-5.852±1.201	-5.308±1.354
E62603	9.038±0.040	8.806±0.192	8.814±0.117	0.000±0.000	-9.038±0.040	-8.714±0.131
